# *Pfeiffer* effect on configurationally labile dyes within ternary complexes with metal ions and enantiopure macrocycles†

**DOI:** 10.1039/d3dt04098d

**Published:** 2024-01-10

**Authors:** Dávid Pál, Jérôme Lacour

**Affiliations:** a Department of Organic Chemistry, University of Geneva Quai Ernest Ansermet 30 CH-1211 Geneva Switzerland jerome.lacour@unige.ch

## Abstract

A configurationally-labile helical dye, 2,4,5,7-tetranitrofluorenone oximate, is used to probe complexes made of enantiopure macrocycles and mono/divalent metal ions. Induced electronic circular dichroism (ECD) and ^1^H NMR responses are amplified at room temperature only in the presence of K^+^ and Na^+^ ions despite larger binding efficiency with alkaline earth metal ions.

## Abstract 2

A labile helical oximate dye is used to probe complexes made of an enantiopure macrocycle and metal ions. Strong induced ECD only occurs in the presence of Na^+^ and K^+^ at 20 °C.

## Graphical abstract

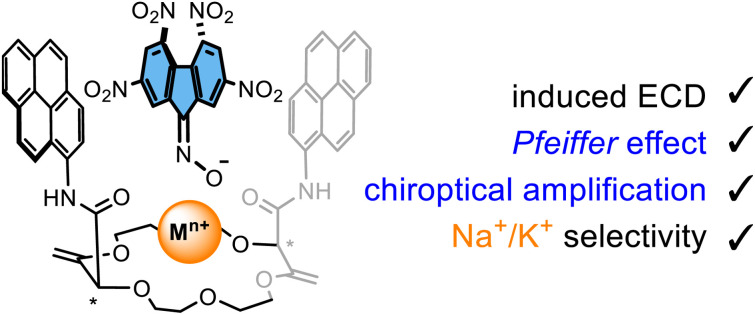

## Introduction

**Fig. 1:**
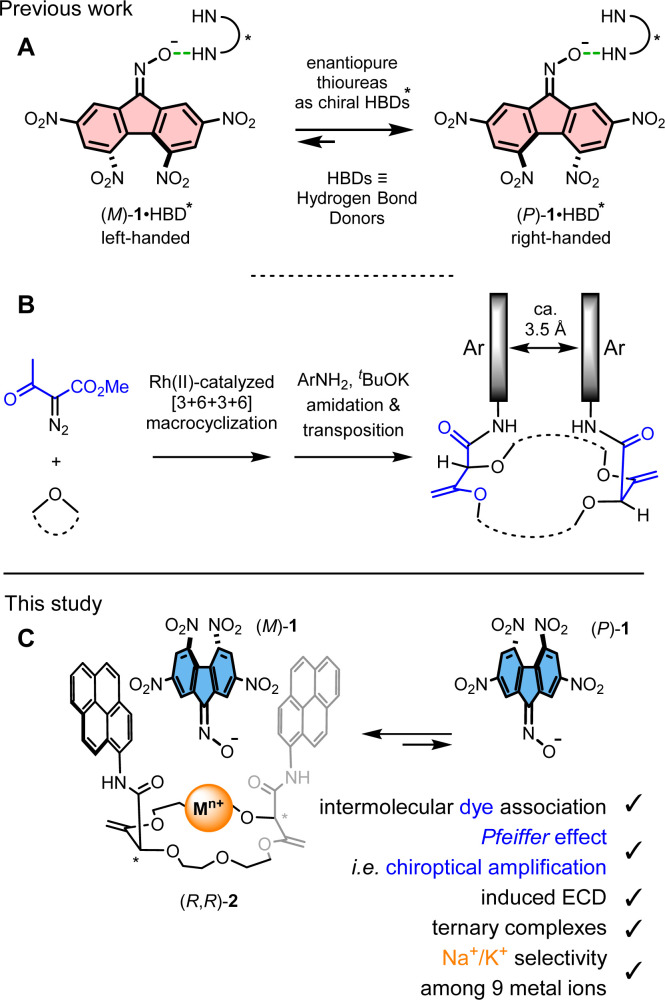
(A) Application of 2,4,5,7-tetranitrofluorenone oximate 1 for chiral HBD recognition. (B) Synthesis of chiral macrocycles of type 2 with close aromatic-aromatic interactions. (C) Chiroptical spectroscopic approach to the evaluation of molecular inclusion by subsequent asymmetric induction; modulation by metal coordination.

Chiral configurationally-labile molecules are abundant in chemistry, from simple moieties such as 1,2-difluoroethane^1^ or *o*,*o*′-disubstituted biaryls,^2^ to dynamic (coordination) complexes,^3^ biomolecules,^4^ and (supramolecular) polymers.^5^ These derivatives that interconvert readily at room temperature between two enantiomeric geometries are most often found as racemic mixtures. However, their association with scalemic or enantiopure partners/environments may lead to supramolecular assemblies that favor the formation of one diastereomeric complex over the other. This asymmetric amplification, called *Pfeiffer* effect,^6^ is usually followed spectroscopically by electronic circular dichroism (ECD),^7,8^ and very high levels of chiral induction can be achieved. Recently, in this context, 2,4,5,7-tetranitrofluorenone oximate 1 was shown to adopt helical (*M*) and (*P*) conformations by repulsion of neighboring nitro groups (Fig. 1A).^9^ Thanks to low energy visible light absorption and effective complexation properties with hydrogen bond donors, a *Pfeiffer* effect could be detected in the presence of chiral bis-thiourea derivatives and characterized by ECD.^9^ Of note, oximate 1 is related to *Newman*'s 2-(((2,4,5,7-tetranitro-9*H*-fluoren-9-ylidene)amino) oxy)propanoic acid (Fig. S1†),^10^ a π-acceptor reagent used prominently for the resolution of electron-rich polyaromatic carbohelicenes.^11^

Previously, independently from the chemistry detailed above, the synthesis of chiral macrocycles of type 2 (Fig. 1B and C) was described using (i) large-scale [3 + 6 + 3 + 6] macrocyclizations of cyclic ethers, *e.g.*, 1,4-dioxane, with α-diazoketoesters under dirhodium catalysis,^12^ and (ii) synchronous amidation/olefin transpositions with aromatic amines and ^*t*^BuOK as reagents.^12*b*^ The unsaturated macrocycles 2 carry two stereogenic centers of same configuration and the racemates can be readily separated as single (*R*,*R*) and (*S*,*S*) enantiomers by chiral stationary phase HPLC.^13^ They also benefit from the preferred conformation induced by the allylic (1,3)-strain that occurs between the stereogenic centers and the adjacent exocyclic C=C double bonds. As a consequence, the two amide arms are oriented perpendicular to the polyether macrocycle and the aromatic moieties reside in close spatial proximity to each other.^13,14^ In fact, several crystallographic structures have been obtained (*e.g.* CCDC 1045592 for 2) and, in every instance, residues like pyrene or perylene present cofacial orientations and short distances in the range of 3.5 Å are observed between the parallel planes of the aromatics (Fig. 1B). Several applications from catalysis to (chir)optical probes and switches have been developed;^12*d*,15^ the switching of properties being often realized by the addition and removal of metal ions. In fact, even in competitive solvents like acetonitrile, complexation in the center of macrocycles 2 of monovalent and divalent metal ions occur with a thermodynamic preference for cations such as Ba^2+^ or Ca^2+^;^16^ a strong binding of monovalent cations occurs in less competitive solvents like dichloromethane.^11*a*^ Also, upon complexation, major conformational changes occur as the exocyclic amide bonds rotate *ca.* 180° and the resulting inward orientation of the C=O bonds helps complex the cations.^17,18^ As a consequence, the aromatic rings carried by the N-atoms move apart from one another, elongating sufficiently the distance between the chromophores to monitor the conformational change (Fig. 1C). In fact, with pyrenes as apical aryl groups, the classical excimer fluorescence is then quenched by the distanciation and only the characteristic emission of (monomeric) pyrene is observed. This allosteric-like behavior impacts strongly the chiroptical properties of the aryl chromophores (*g*_lum_ varying from >10^−2^ to zero). Also and of importance for the current study, despite many attempts, in the absence or presence of metal ions, inclusion of organic guest molecules within the cleft formed by the two aromatic amide residues has never been achieved. This somewhat puzzling observation occurs even when classical neutral electron-poor aromatic residues are added to solutions of macrocycles of type 2. Herein, to induce this type of missing intermolecular inclusion interactions, ternary situations are implemented. The addition of enantiopure bispyrene-containing 2 to anionic electron-poor tetranitrofluorenone oximate 1 is studied in the presence of alkali or alkaline earth metal ions that can act as electrostatic glue to bring all elements together. Results indicate that, under these conditions, oximate 1 becomes a subtle chiroptical probe of the stereochemical modifications inside the macrocyclic host 2, in the direct environment of the metal ion at the core. In acetonitrile, a clear dichotomy can be found in UV–vis spectroscopy for the binding of monovalent *vs.* divalent ions. However, only chiroptical studies provide an efficient method to distinguish cations in the Li^+^ to Cs^+^ series. In fact, in the presence of 1, strong induced *Cotton* effects appear at low energy (450–650 nm) only in the cases of Na^+^ and K^+^. The asymmetric amplification is rationalized by the predominant formation of diastereomeric ternary complexes made of the metal ions, enantiopure macrocycle (*R*,*R*)- or (*S*,*S*)-2, and oximate 1 with preferred (induced) (*M*)- or (*P*)-configurations, respectively.

## Results and discussion

**Fig. 2:**
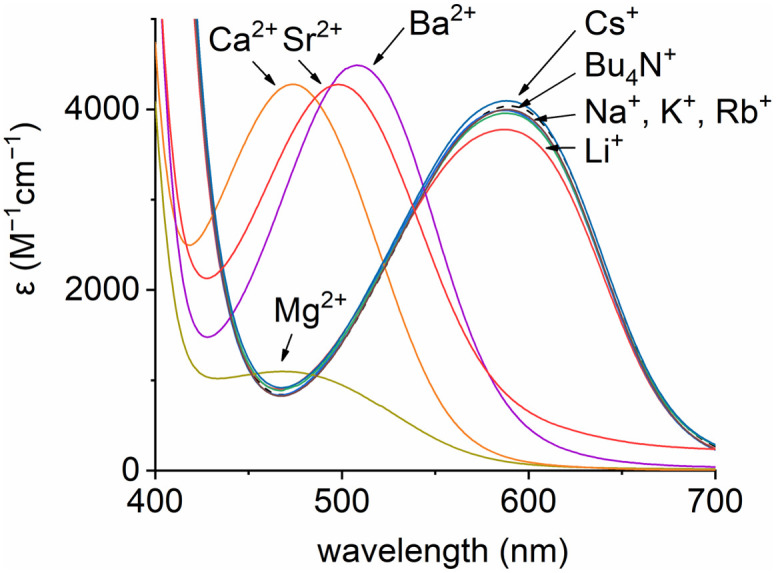
Absorption spectra of 1 (200 μM) with different counterions in MeCN.

**Fig. 3:**
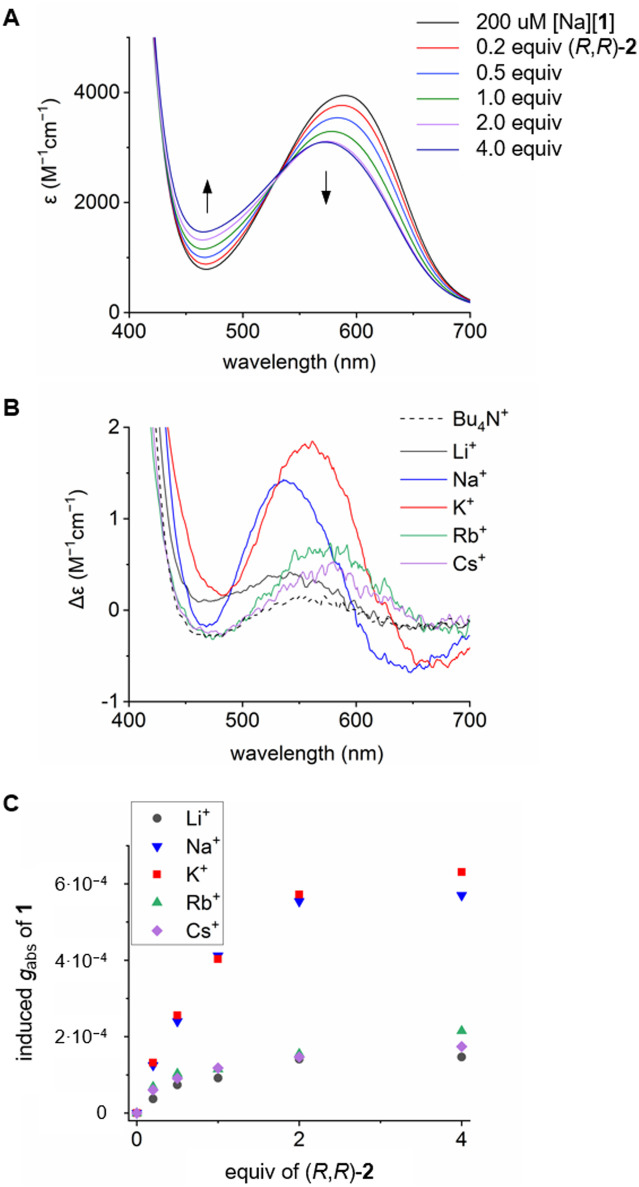
(A) Titration of [Na][1] (200 μM) with (*R*,*R*)-2 in MeCN, (B) ECD spectra of 1 (200 μM) with different alkali and Bu_4_N^+^ counterions in the presence of 4.0 equiv. of (*R*,*R*)-2, (C) induced *g*_abs_ of 1 as a function of (*R*,*R*)-2 quantity, alkali metal ion-dependence.

To investigate the roles of the various components within the ternary assembly, *i.e.* oximate 1/chiral macrocycle 2/metal ion M^*n*+^, it was necessary to evaluate first the interactions of these elements in one-by-one (binary) associations. For instance, the absorption spectrum of oximate 1 was measured in the presence of nine mono- and divalent metal ions (Li^+^, Na^+^, K^+^, Rb^+^, Cs^+^, Mg^2+^, Ca^2+^, Sr^2+^, Ba^2+^) and also organic Bu_4_N^+^; the tetrabutylammonium cation, unable to bind to the crown ether fragment of the macrocycle, serving as control reference for this and latter studies. Results are displayed in Fig. 2. In the case of divalent alkaline earth metal ions bearing higher electrostatic factors,^16^ the low energy band of 1 was blue-shifted, reminiscent of the interaction of 1 with hydrogen-bond donors.^9^ However and of importance, alkali metal ions and Bu_4_N^+^ did not cause any spectral shift as absorptions remained centered at 590 nm; this opening the door to ECD studies of chiral induction at rather low energy. Then, enantiopure macrocycle 2 was titrated with inert Bu_4_N^+^ as well as alkali and alkaline earth metal perchlorate salts. As expected,^16^ formation of 1 : 1 complexes occurred with the metal ions. The ECD spectra of (*R*,*R*)-2 and (*S*,*S*)-2 underwent significant changes upon addition of each metal ion in acetonitrile at high energy (200–400 nm) (Fig. S4–S6†).^19^ Overall, stronger effect can be observed in the divalent series; *Cotton* effects for certain transition(s) switch from positive to negative values, or *vice versa*. In the alkali series, such a switching behavior is prevalent only with Na^+^ and K^+^. These switches indicate strong variations of the stereochemical environment of the metal ions, with a lot of subtle differences from one system to the next. Finally, [Bu_4_N][1] was mixed with 4.0 equivalents of (*R*,*R*)-2; the UV–vis and ECD spectra being monitored in the absence of metal ions (Fig. S7†). From 450 to 700 nm, little variation was found in the visible region as the pyrene units of macrocycle 2 do not absorb at low energy and only a very weak induction was noticed for 1 in ECD (dashed black curve, Fig. 3B).

With these results in hand, three-component (ternary) systems were studied by UV–vis and ECD spectroscopies (Fig. 3 and Fig. S8–S10†). First, and somewhat surprisingly, coordination of (*R*,*R*)-2 with [Ba]_½_[1] salt did not result in any noticeable induction in ECD (Fig. S10†) even though Ba^2+^ forms a particularly stable 1 : 1 complex with 2 (log *β* 6.28 in acetonitrile at 293 K).^16^ Gratifyingly, using macrocycle (*R*,*R*)-2 and alkali metal ions instead, stereoinduction onto configurationally labile 1 occurred – the series from Li^+^ to Cs^+^ presenting major differences though. The smallest (Li^+^) and the two largest cations (Rb^+^, Cs^+^) led to little effects in absorption (Fig. S8†) and ECD (Fig. S9†) with weak positive and monosignate *Cotton* effects around 540–580 nm. However, with Na^+^ and K^+^ ions, more intense spectral changes occurred and bisignate signals were observed in the region of the π → π* and n → π* transitions of 1; the signals induced by the presence of Na^+^ being blue-shifted compared to K^+^ (Fig. 3A and B). Using (*R*,*R*)-2, ECD signals of 1 are positive and negative at 538/560 and 648/670 nm in the presence of Na^+^/K^+^, respectively, indicating a preferred (*M*) over (*P*) configuration for the configurationally labile dye 1 (Fig. 1C).^9^

At that stage, it was essential to confirm, by an independent method, the formation of the ternary complex between 1, 2 and Na^+^, and obtain additional structural information on the binding, and evidence of a potential inclusion of the oximate between the aromatic pyrene amide arms in particular. It was performed by ^1^H NMR titration of 2 with [Na][1] (Fig. S14†), and somewhat contradicting evidences seemed to be obtained initially.^20,21^ On one side, all aromatic signals of complexed oximate 1 broadened and appeared at substantially lower frequencies than those of dissociated (free) oximate 1. At the same time, for macrocycle 2, two protons of each pyrene units exhibited marked shifts toward lower frequencies (Δ*δ* −0.58 and −0.38 ppm). This experiment favors direct contacts between electron-poor 1 and the electron-rich pyrene units of 2; the type of contacts looked-for at the onset of the study. The geometry represented in Fig. 1 (C, left) corresponds best to the structural data gathered at room temperature.

**Fig. 4:**
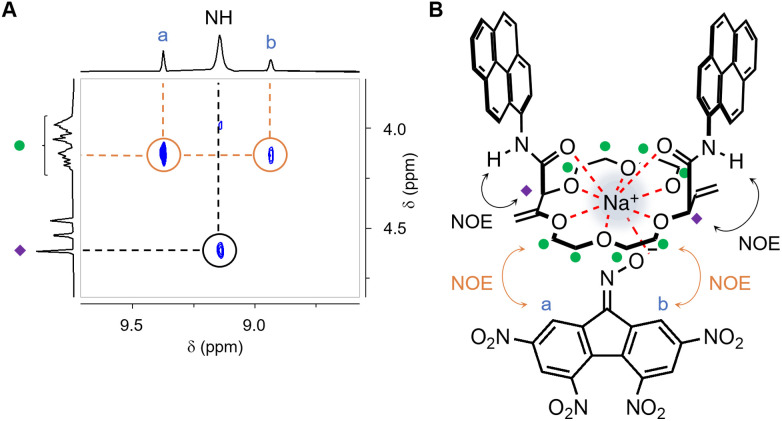
(A) Partial NOESY (MeCN-*d*_3_, 500 MHz, 343 K, *d*_8_ = 500 ms) of 1 : 2 mixture of [Na][1] (1 mM) and 2 (2 mM). (B) Plausible higher temperature ternary complex.

On the other side, at 343 K, to benefit from a sharpening of the signals at elevated temperature, 2D NOESY spectra of 1 : 2 mixtures of [Na][1] (1 mM) and 2 (2 mM) were performed (Fig. S18†). NOE correlations can be observed between the amide NH and α-allylic CH protons, NH ↔ ◆, confirming the outward orientation of the NH moieties. This validates the most-likely coordination of Na^+^ into the center of the macrocycle that attracts the Lewis basic carbonyl groups inward. Furthermore, and of importance, other exchange cross-peaks, a ↔ • and b ↔ •, are observed and depicted in Fig. 4. These signals demonstrate a proximity of the protons adjacent to C=N–O^−^ group of 1 to that of the crown ether ring bound to the sodium salt; the other protons of 1 did not afford any such exchange signals. For the pyrene units, close contacts with oximate 1 (NMR shielding) cannot be monitored anymore at that elevated temperature. In other words, the combined NMR experiments suggest that, at 343 K, 1 interacts with the ring of 2, away from the amide aromatic arms.

**Fig. 5:**
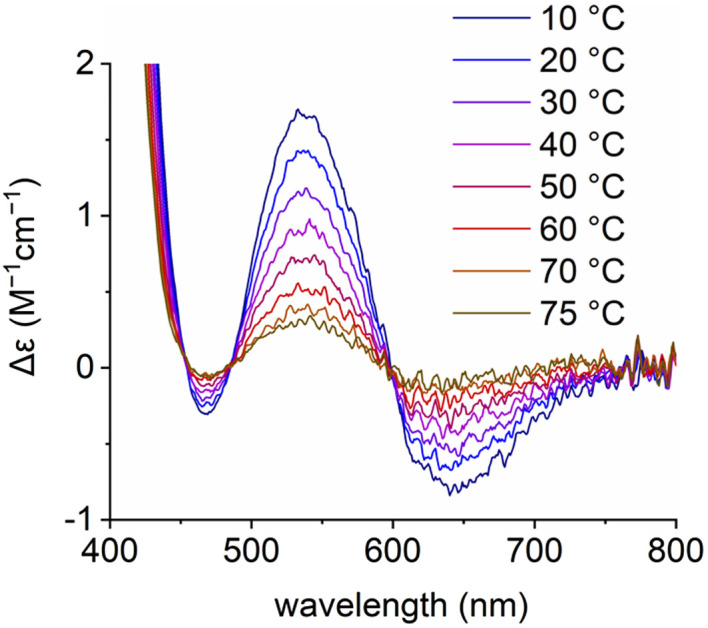
ECD spectra of [Na][1] (200 μM) in the presence of 4.0 equiv. of (*R*,*R*)-2 at various temperatures (from 10 °C to 75 °C) in MeCN.

This dual mode of interactions for the ternary complex between 1, 2 and Na^+^ favoring (i) the intra-cleft inclusion of 1 at room temperature (Fig. 1C) and (ii) a direct electrostatic attraction on the polyether side of 2 between the C=N–O^−^ moiety and Na^+^ (Fig. 4) at 70 °C, needed to be confirmed. Hypothesizing that the supramolecular chiral induction of (*R*,*R*)-2 over interconverting (*M*) and (*P*) conformations of 1 would be preferred in the low-temperature inclusion structure, care was thus taken to perform a variable temperature (VT) ECD experiment from 10 to 75 °C (Fig. 5). A strong modulation was noticed and induced *Cotton* effects become much weaker at elevated temperatures (Δ*ε* 1.67 to 0.31 M^−1^ cm^−1^). The low asymmetric induction at 75 °C is interpreted as the consequence of the geometry depicted in Fig. 4B in which the twisted helical backbone of 1 is positioned away from the chiral macrocycle.^22^ Finally, the ECD spectra obtained at high temperature coincide effectively with that of ternary complexes made of 1, 2 and alkaline earth metal ions M^2+^ at 20 °C. It is thus likely that the (second) geometry favoring the complexation of oximate 1 from the polyether side is also favored in the case of Ba^2+^.^23^

## Experimental

### Synthesis and characterization

Starting materials were purchased from Sigma-Aldrich unless otherwise noted. Compounds [Bu_4_N][1], [Na][1], and 2 were prepared as reported in the literature. Lithium perchlorate (Fluka), sodium perchlorate monohydrate (Fluka), potassium perchlorate (Acros Organics), rubidium perchlorate (abcr), cesium perchlorate (Sigma-Aldrich), magnesium perchlorate (Fluka), calcium perchlorate tetrahydrate (Acros Organics), strontium perchlorate trihydrate (Thermo Scientific), and barium perchlorate (Acros Organics) were purchased from commercial sources and used without further purification. Enantiomers of *rac*-2 were separated by HPLC using a Daicel Chiral Technologies CHIRALPAK® IG (inner diameter × length; particle size: 10 mm × 250 mm; 5 μm) semi-preparative column as chiral stationary phase and CH_2_Cl_2_–MeCN 70 : 30 (0.1% Et_2_NH) as eluent (2.0 mL min^−1^ flow rate, 25 °C). 23 mg of *rac*-2 dissolved in CH_2_Cl_2_ (1.5 mL), inj.: 500 μL. Ratios of solvents for the eluents are given in volumes (mL mL^−1^). NMR spectra were measured using a Bruker Avance III HD Nanobay 400 MHz spectrometer equipped with a 5 mm CPP BBO probe, or using a Bruker Avance III 500 MHz spectrometer equipped with a 5 mm CPP DCH ^13^C–^1^H/D helium-cooled cryogenic probe. NMR solvent: MeCN-*d*_3_ (Apollo Scientific). All signals were internally referenced to the solvent residue (MeCN-*d*_3_: 1.940 ppm for ^1^H NMR).

### UV–vis and electronic circular dichroism measurements

UV–visible spectra were taken using a Jasco V-650 spectrophotometer equipped with a programmable temperature control system. Electronic circular dichroism (ECD) spectra were measured using a Jasco J-815 spectrometer equipped with a Peltier cuvette holder and a programmable temperature control system with the following parameters: 200 nm min^−1^ scan speed, 1 nm slit width, 1 s integration time, 10 accumulations. Temperature was set to 20 °C unless otherwise noted. All spectra were baseline corrected by subtraction of the solvent spectrum. All spectra were recorded in analytical grade acetonitrile. Quartz cuvettes with path length of 1 cm were used. Software: Spectra Manager.

## Conclusions

Ion pairs of configurationally labile helical oximate 1 were applied to probe the stereochemical environment of an enantiopure diamide macrocyclic ligand and the inclusion of the dye within the two pyrene subunits. The *Pfeiffer* effect, visualized by the induced ECD, was highly dependent on metal coordination and on temperature. At 20 °C, only K^+^ and Na^+^ complexes led to strong diastereoselective amplification contrary to divalent cations, despite their higher binding affinity to the inner core of the macrocycle. At higher temperature, an alternative ternary complex geometry must be proposed to account for the ^1^H NMR spectroscopy data and the sharp loss of asymmetric induction by ECD. The use of the *Pfeiffer* effect remains thus an alternative yet valuable methodological approach for structural analysis of supramolecular assemblies.

## Author contributions

D. P. and J. L. conceived the original idea. D. P. synthesized the compounds, designed, and performed the experiments, analyzed, and visualized data. J. L. oversaw the project. D. P. and J. L. discussed the results and wrote the final manuscript.

## Data availability statement

The data that support the findings of this study are openly available in yareta.unige.ch at https://doi.org/10.26037/yareta:apv2zpswabbsxfsmpvhbulnpni. It will be preserved for 10 years.

## Conflicts of interest

There are no conflicts to declare.

## Supplementary Material

DT-053-D3DT04098D-s001
