# Supplementary material for: Pfeiffer effect on configurationally labile dyes within ternary complexes with metal ions and enantiopure macrocycles
Source: Dalton Trans. 2024 Jan 10;53(6):2665–9. doi: 10.1039/d3dt04098d (PMC10845008; doi:10.1039/d3dt04098d)
Supplement: DT-053-D3DT04098D-s001 [file DT-053-D3DT04098D-s001.pdf]

Electronic Supplementary Material (ESI) for Dalton Transactions.
This journal is © The Royal Society of Chemistry 2024

Pfeiffer effect on Configurationally Labile Dyes within Ternary Complexes with Metal Ions and Enantiopure Macrocycles

Dávid Pál and Jérôme Lacour*

Department of Organic Chemistry, University of Geneva, 30 quai Ernest Ansermet, CH-1211 Geneva, Switzerland

Supporting Information

Contents

1. Overview of compounds S3
2. Experimental methods S4
  2.1. Synthesis and characterization S4
  2.2. UV–vis and electronic circular dichroism measurements S4
3. Synthesis of the compounds S5
  3.1. Oximate salts S5
    3.1.1. Potassium 2,4,5,7-tetranitro-9H-fluoren-9-one oximate [K][**1**] S5
  3.2. Macrocycles S8
4. UV–vis and electronic circular dichroism (ECD) studies S9
  4.1. UV–vis and ECD spectra of enantiopure **2** with Bu_4N^+ ion (control experiments) S9
  4.2. UV–vis and ECD titration of (*R*,*R*)-**2** and (*S*,*S*)-**2** with alkali metal ions S10
  4.3. UV–vis and ECD titration of (*R*,*R*)-**2** and (*S*,*S*)-**2** with alkaline earth metal ions S12
  4.4. UV–vis and ECD titration of [Bu_4N][**1**] with (*R*,*R*)-**2** S13
  4.5. UV–vis and ECD titration of **1** with (*R*,*R*)-**2** in the presence of alkali metal ions S14
  4.6. UV–vis and ECD titration of **1** with (*R*,*R*)-**2** in the presence of Ba^{2+} ions S16
5. ^{1}H NMR experiments S17
6. Temperature-dependent equilibrium between ternary complexes S23
7. References S24

1. Overview of compounds

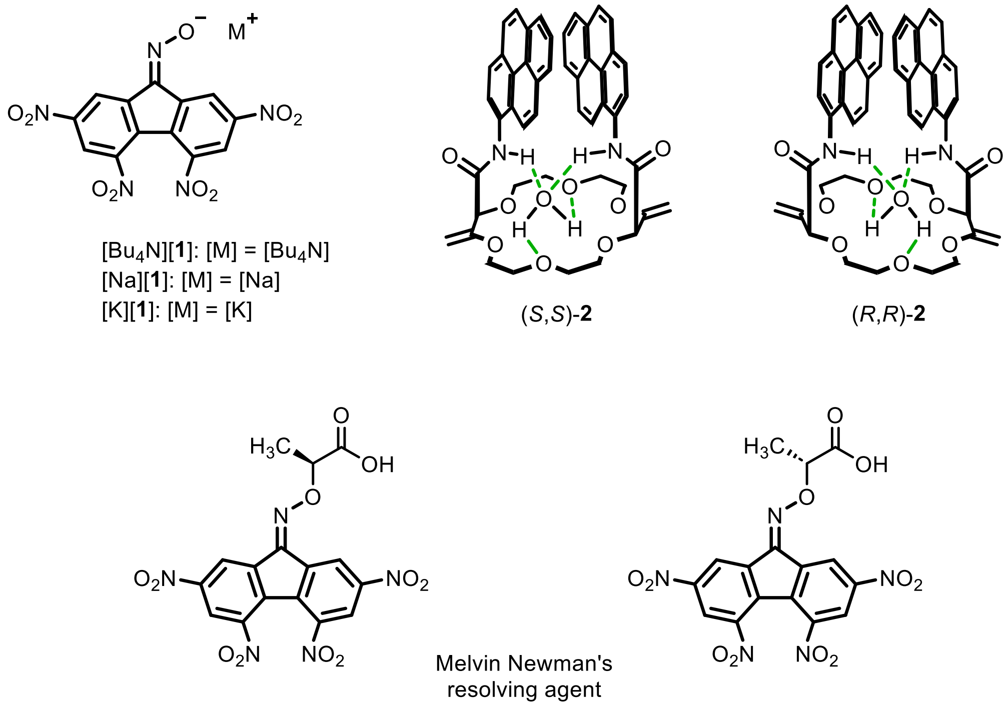

(S)- and (R)-2-(((2,4,5,7-tetranitro-9H-fluoren-9-ylidene)amino)oxy)propanoic acid

Figure S1.

2. Experimental methods

2.1. Synthesis and characterization

Starting materials were purchased from Sigma–Aldrich unless otherwise noted. Compounds [Bu_4N][**1**],[S1] [Na][**1**],[S1] and **2**[S2] were prepared as reported in the literature. Lithium perchlorate (Fluka), sodium perchlorate monohydrate (Fluka), potassium perchlorate (Acros Organics), rubidium perchlorate (abcr), cesium perchlorate (Sigma–Aldrich), magnesium perchlorate (Fluka), calcium perchlorate tetrahydrate (Acros Organics), strontium perchlorate trihydrate (Thermo Scientific), and barium perchlorate (Acros Organics) were purchased from commercial sources and used without further purification. Enantiomers of *rac*-**2** were separated by HPLC using a Daicel Chiral Technologies CHIRALPAK® IG (inner diameter × length; particle size: 10 mm × 250 mm; 5 µm) semi-preparative column as chiral stationary phase and CH_2Cl_2–MeCN 70:30 (0.1% Et_2NH) as eluent (2.0 mL/min flow rate, 25 °C). 23 mg of *rac*-**2** dissolved in CH_2Cl_2 (1.5 mL), inj.: 500 µL. Ratios of solvents for the eluents are given in volumes (mL/mL). NMR spectra were measured using a Bruker Avance III HD Nanobay 400 MHz spectrometer equipped with a 5 mm CPP BBO probe, or using a Bruker Avance III 500 MHz spectrometer equipped with a 5 mm CPP DCH ^{13}C-^{1}H/D helium-cooled cryogenic probe. NMR solvent: MeCN-d_3 (Apollo Scientific). All signals were internally referenced to the solvent residue (MeCN-d_3: 1.940 ppm for ^{1}H NMR).

2.2. UV–vis and electronic circular dichroism measurements

UV–visible spectra were taken using a Jasco V-650 spectrophotometer equipped with a programmable temperature control system. Electronic circular dichroism (ECD) spectra were measured using a Jasco J-815 spectrometer equipped with a Peltier cuvette holder and a programmable temperature control system with the following parameters: 200 nm/min scan speed, 1 nm slit width, 1 sec integration time, 10 accumulations. Temperature was set to 20 °C. All spectra were baseline corrected by subtraction of the solvent spectrum. All spectra were recorded in analytical grade acetonitrile. Quartz cuvettes with path length of 1 cm were used. Software: Spectra Manager.

3. Synthesis of the compounds

3.1. Oximate salts

A straightforward synthesis of oximate salts was carried out as outlined in Scheme S1. Condensation of tetranitrofluorenone **3**[S3] with hydroxylamine hydrochloride afforded oxime **4**.[S4] Then, various ion pairs were prepared by quantitative reactions with hydroxide/methoxide bases to afford the sodium, potassium and tetrabutylammonium salts, namely [Na][**1**],[S1] [K][**1**] and [Bu_4N][**1**].[S1]

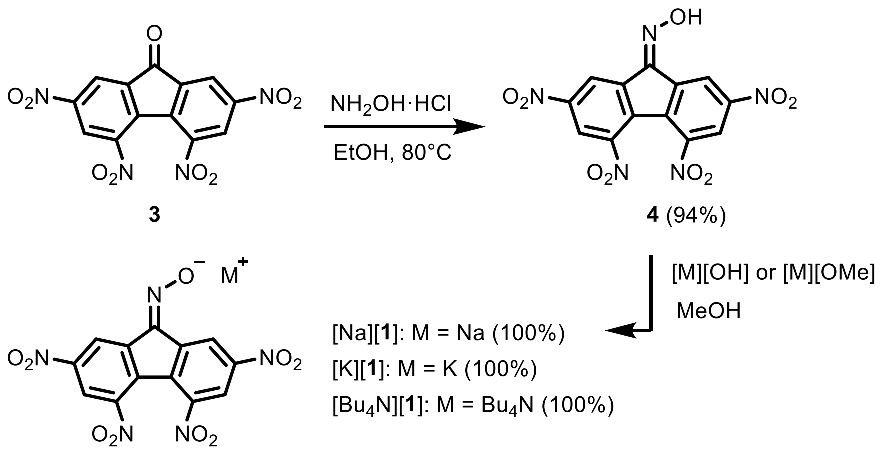

Scheme S1. Synthesis of salts [Na][**1**], [K][**1**], and [Bu_4N][**1**].

3.1.1. Potassium 2,4,5,7-tetranitro-9H-fluoren-9-one oximate [K][1]

To a solution of 2,4,5,7-tetranitro-9*H*-fluoren-9-one oxime (**4**, 20.0 mg, 0.0533 mmol) in methanol (1.5 mL) was added a 0.1 N methanolic solution of potassium methoxide (0.0533 mmol, 0.533 mL). The resulting dark red mixture was stirred at room temperature for 2 hours then the volatile components were removed under reduced pressure to obtain dark red crystals. Yield: 22 mg (100%) dark red (burgundy) crystals. M. p.: 290 °C (decomposition). ^{1}H NMR (MeCN-d_3, 500 MHz, 298 K) δ (ppm): 9.77 (d, J = 2 Hz, 1H), 9.58 (d, J = 2 Hz, 1H), 8.57 (d, J = 2 Hz, 1H), 8.55 (d, J = 2 Hz, 1H). ^{13}C NMR (MeCN-d_3, 125 MHz, 298 K) δ (ppm): 152.2, 149.7, 146.9, 146.9, 145.9, 145.8, 127.0, 122.4, 119.8, 119.8, 119.0, 115.1, 114.2. IR (solid) ν_{max} (cm^{-1}): 3091, 1573, 1518, 1451, 1336, 1321, 1234, 1162, 1134, 1097, 1062, 1015, 930, 845, 726. HR-MS (ESI–) calculated for $C_{13}H_4N_5O_9^-$ [M]$^-$ (*m/z*) 374.0009, found 374.0005.

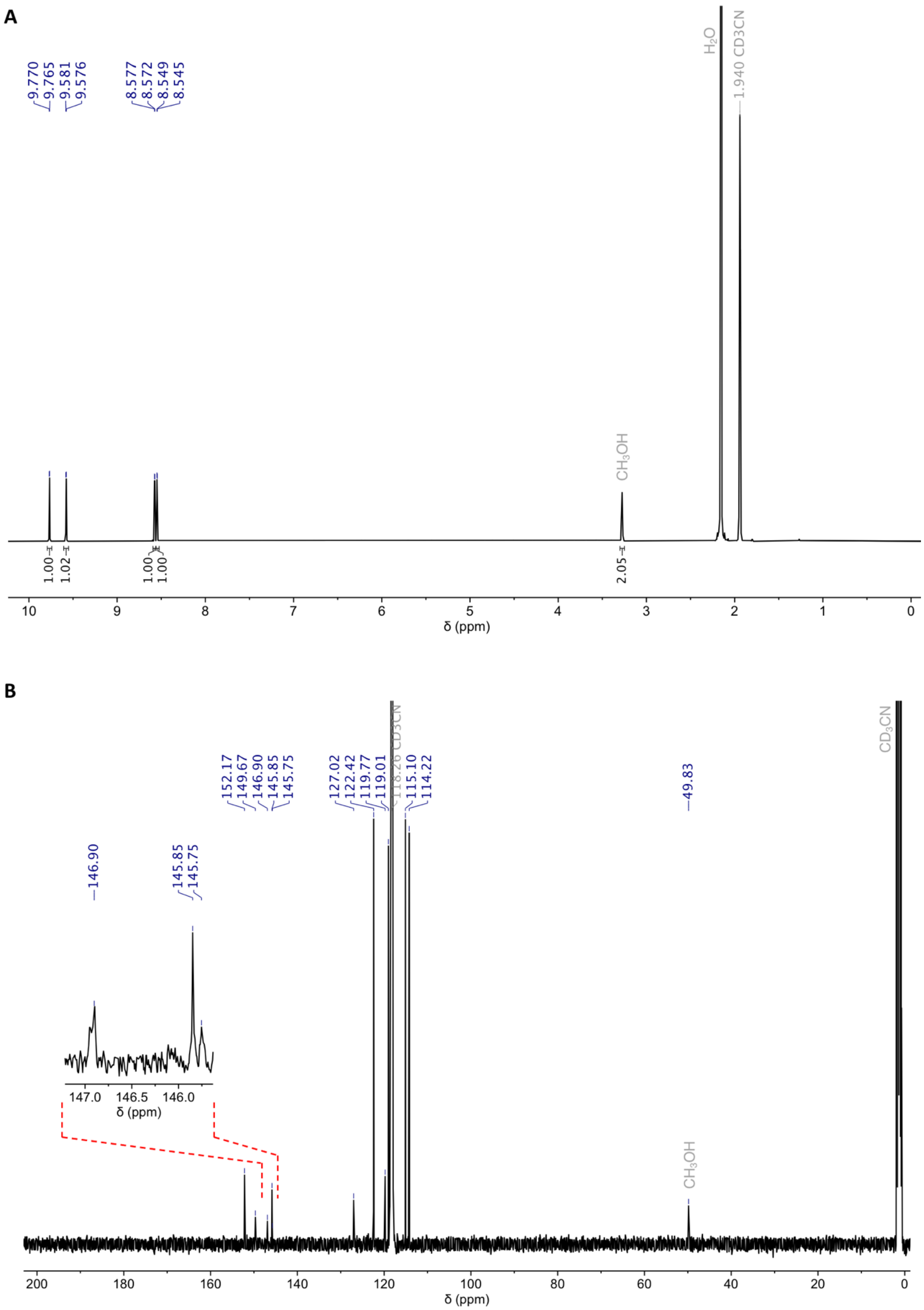

Figure S2. A) ^{1}H NMR (MeCN-d_3, 500 MHz, 298 K) and **B)** ^{13}C NMR (MeCN-d_3, 125 MHz, 298 K) spectra of compound [K][**1**].

3.2. Macrocycles

The two-step synthesis of constrained polyether macrocycle *rac*-**2** was developed in our group (Scheme S2). The rhodium(II)-catalyzed 3+6+3+6 reaction of α-diazo-β-ketoester **5** and 1,4-dioxane enabled the efficient large-scale preparation of macrocycle **6**.[S5,S6] Chiral 18-crown-6 ether-based *rac*-**2** was then formed through a *syn* diastereoselective (d.r. > 49:1) double and tandem amidation-olefin transposition process of diester **6**.[S2,S7] The enantiomers of *rac*-**2** were separated by semi-preparative CSP-HPLC.

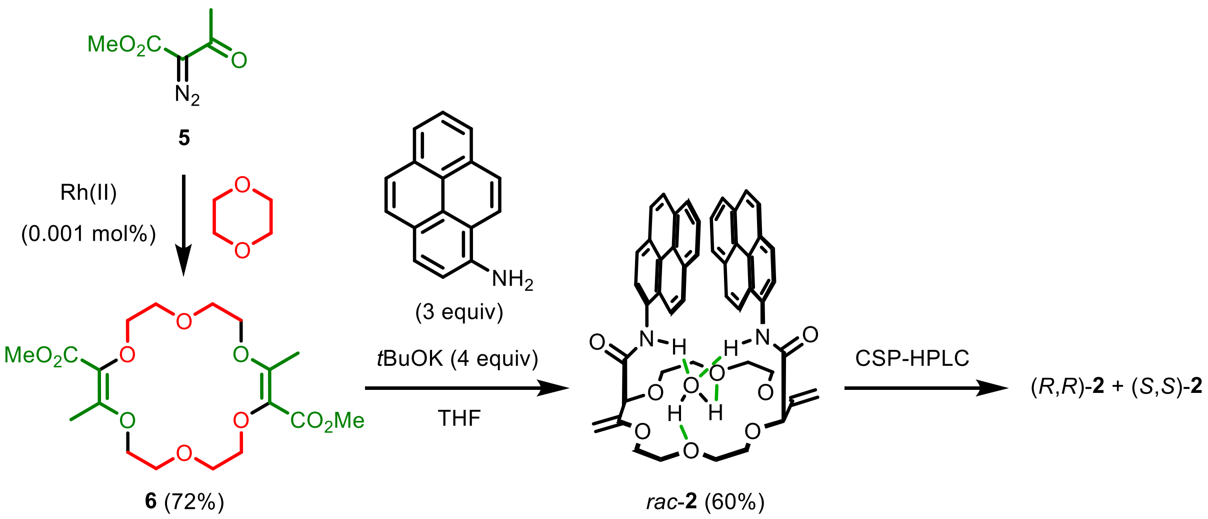

Scheme S2. Synthesis of functionalized macrocycle **2**.

4. UV–vis and electronic circular dichroism (ECD) studies

Perchlorate salts were used in the UV–visible and ECD studies.

4.1. UV–vis and ECD spectra of enantiopure **2** with Bu_4N^+ ion (control experiments)

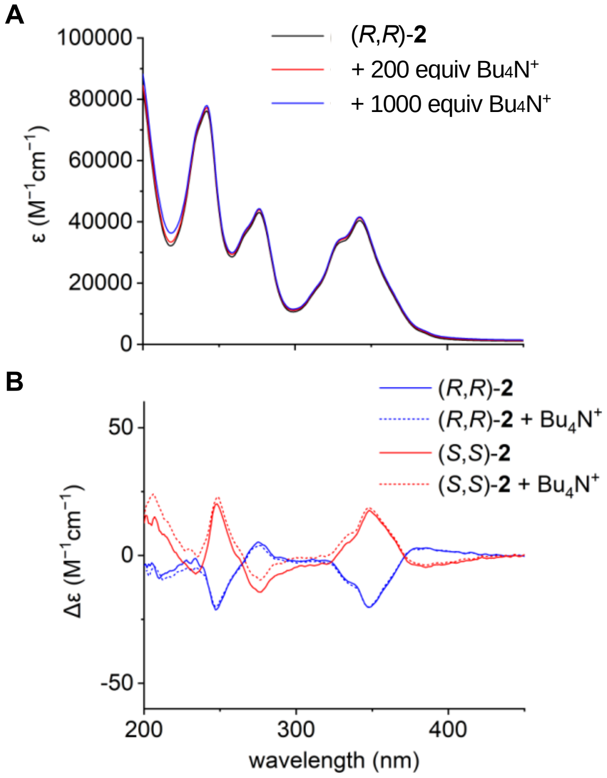

Figure S3. A) UV–vis and **B)** ECD spectra of enantiopure **2** (20 µM), and the spectra in the presence of 1000 equiv Bu_4N^+ in MeCN.

4.2. UV–vis and ECD titration of (*R*,*R*)-**2** and (*S*,*S*)-**2** with alkali metal ions

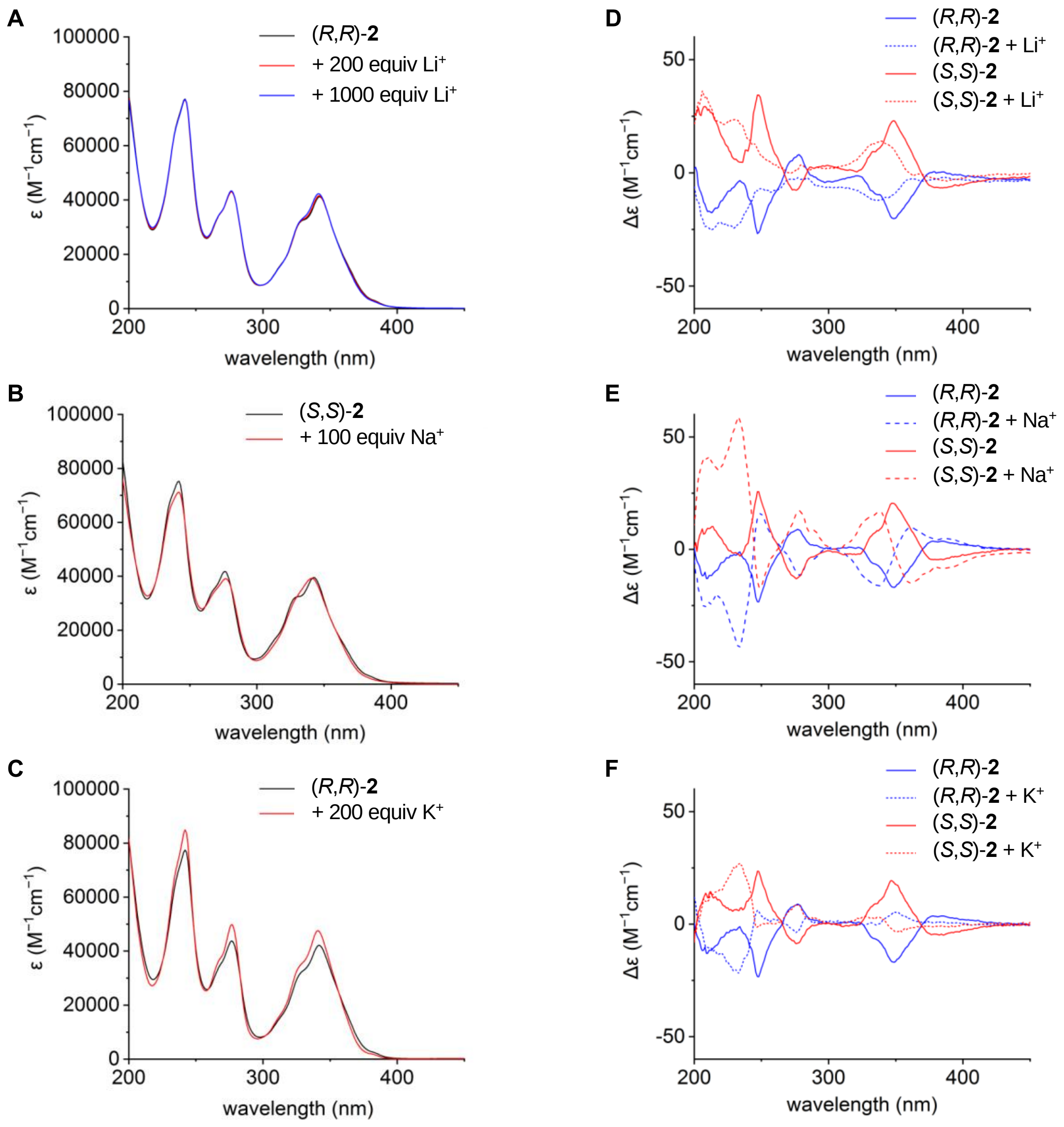

Figure S4. UV–vis (**A**–**C**) and ECD (**D**–**F**) spectra of (*R*,*R*)-**2** (20 µM), (*S*,*S*)-**2** (20 µM), and their spectra in the presence of Li^+, Na^+ or K^+ in MeCN.

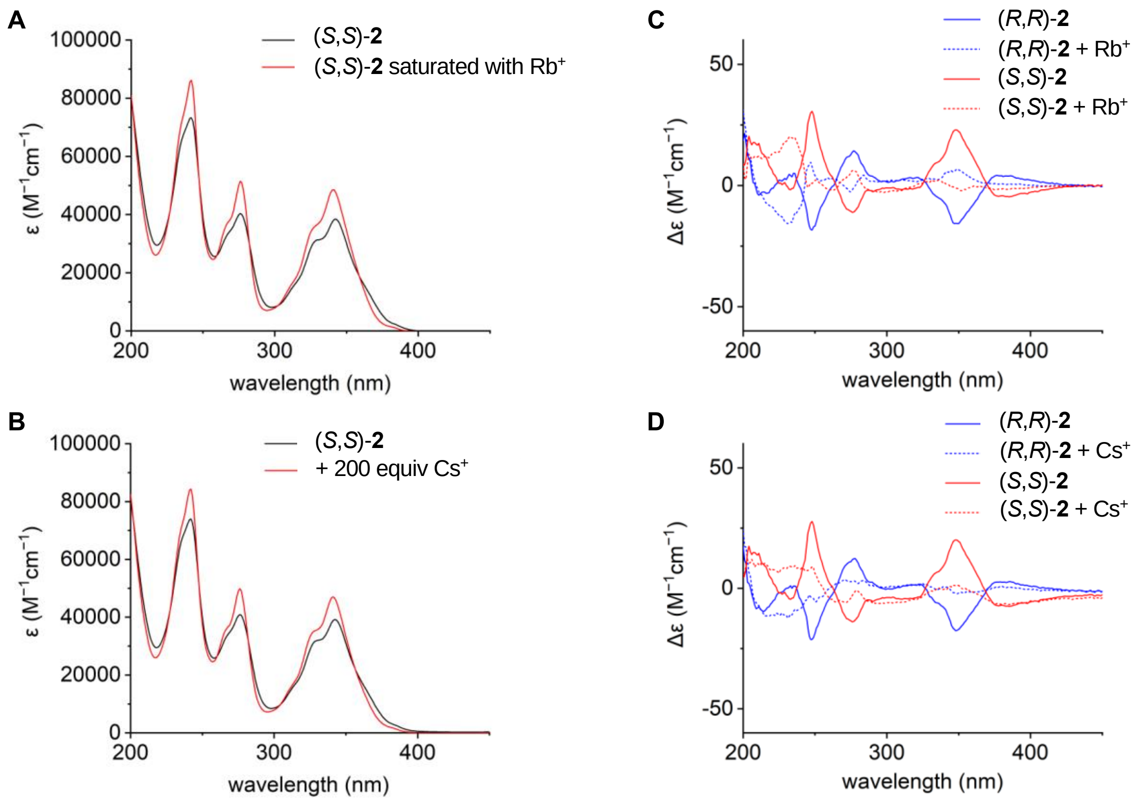

Figure S5. UV–vis (**A** and **B**) and ECD (**C** and **D**) spectra of (*R*,*R*)-**2** (20 µM), (*S*,*S*)-**2** (20 µM), and their spectra in the presence of Rb^+ (solution saturated with $RbClO_4$) or Cs^+ in MeCN.

4.3. UV–vis and ECD titration of (*R*,*R*)-**2** and (*S*,*S*)-**2** with alkaline earth metal ions

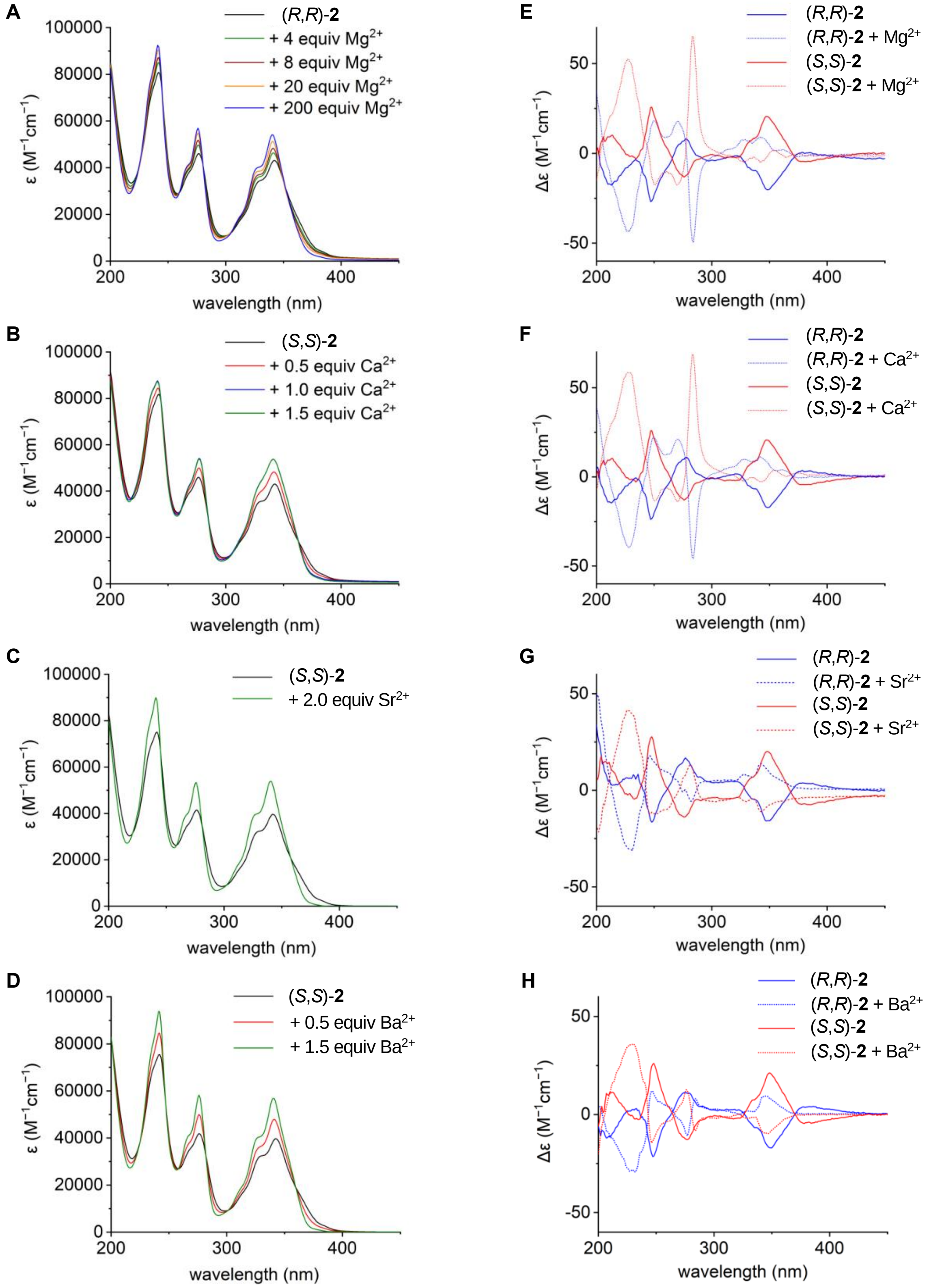

Figure S6. UV–vis (**A**–**D**) and ECD (**E**–**H**) spectra of (*R*,*R*)-**2** (20 μM), (*S*,*S*)-**2** (20 μM), and their spectra in the presence of Mg^{2+}, Ca^{2+}, Sr^{2+} or Ba^{2+} in MeCN.

4.4. UV–vis and ECD titration of [Bu$_4$N][**1**] with (*R*,*R*)-**2**

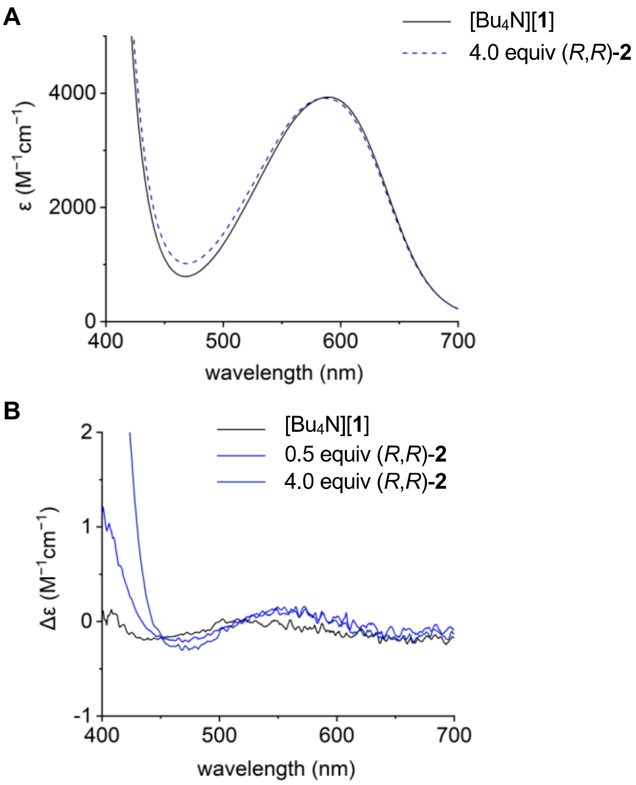

Figure S7. UV–vis (**A**) and ECD (**B**) spectra of [Bu$_4$N][**1**] (200 μM), and the spectra in the presence of (*R*,*R*)-**2** in MeCN.

4.5. UV–vis and ECD titration of **1** with (*R*,*R*)-**2** in the presence of alkali metal ions

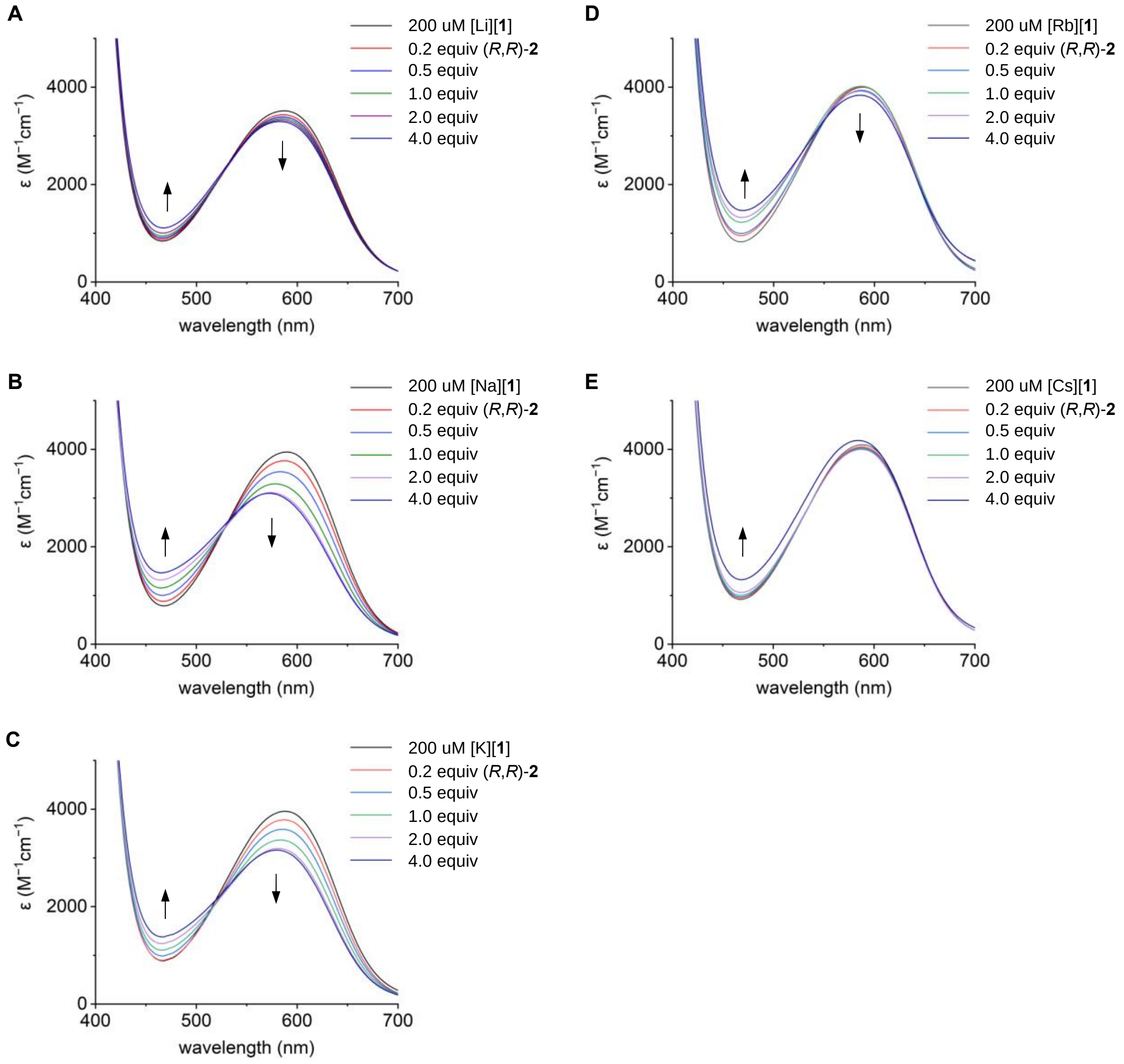

Figure S8. Series of absorption spectra upon titration of **A)** [Li][**1**], **B)** [Na][**1**], **C)** [K][**1**], **D)** [Rb][**1**] or **E)** [Cs][**1**] (200 µM) with (*R*,*R*)-**2** in MeCN.

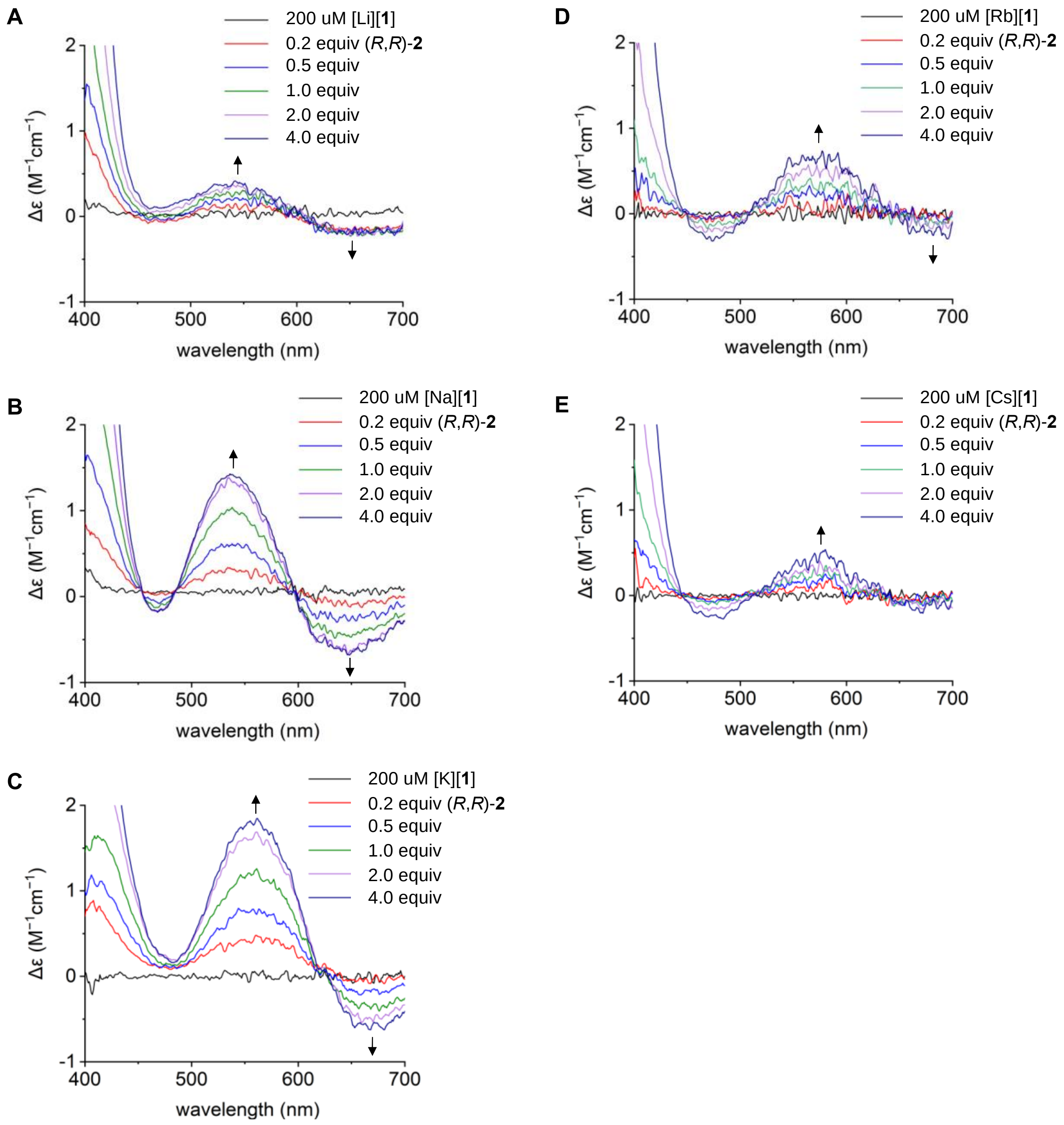

Figure S9. Series of ECD spectra upon titration of **A)** [Li][**1**], **B)** [Na][**1**], **C)** [K][**1**], **D)** [Rb][**1**] or **E)** [Cs][**1**] (200 μM) with (*R*,*R*)-**2** in MeCN.

4.6. UV–vis and ECD titration of **1** with (*R*,*R*)-**2** in the presence of Ba^{2+} ions

Note: to compare ε molar extinction coefficients and Δε differential molar extinction coefficients globally, values are given for formula $[Ba]_{½}[\mathbf{1}]$.

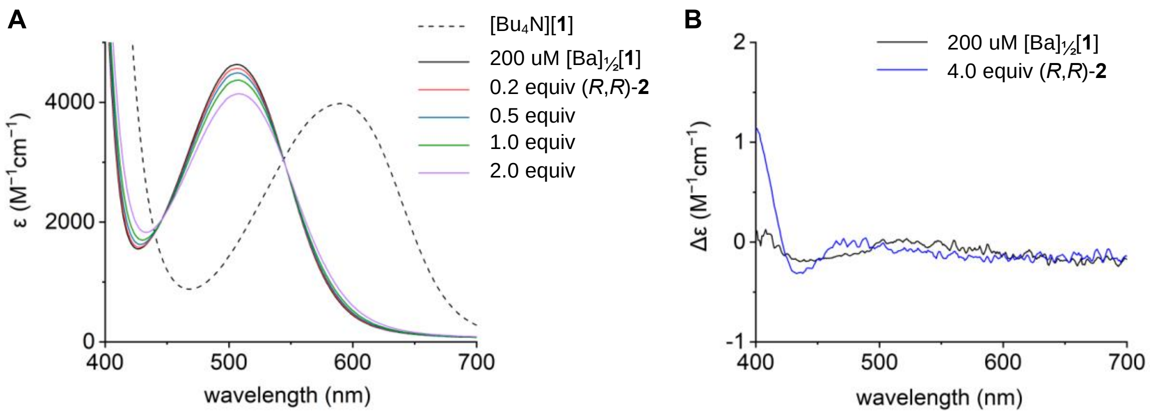

Figure S10. A) Series of absorption spectra, and **B)** ECD spectra upon titration of $[Ba]_{½}[\mathbf{1}]$ (200 µM) with (*R*,*R*)-**2** in MeCN.

5. ^{1}H NMR experiments

A

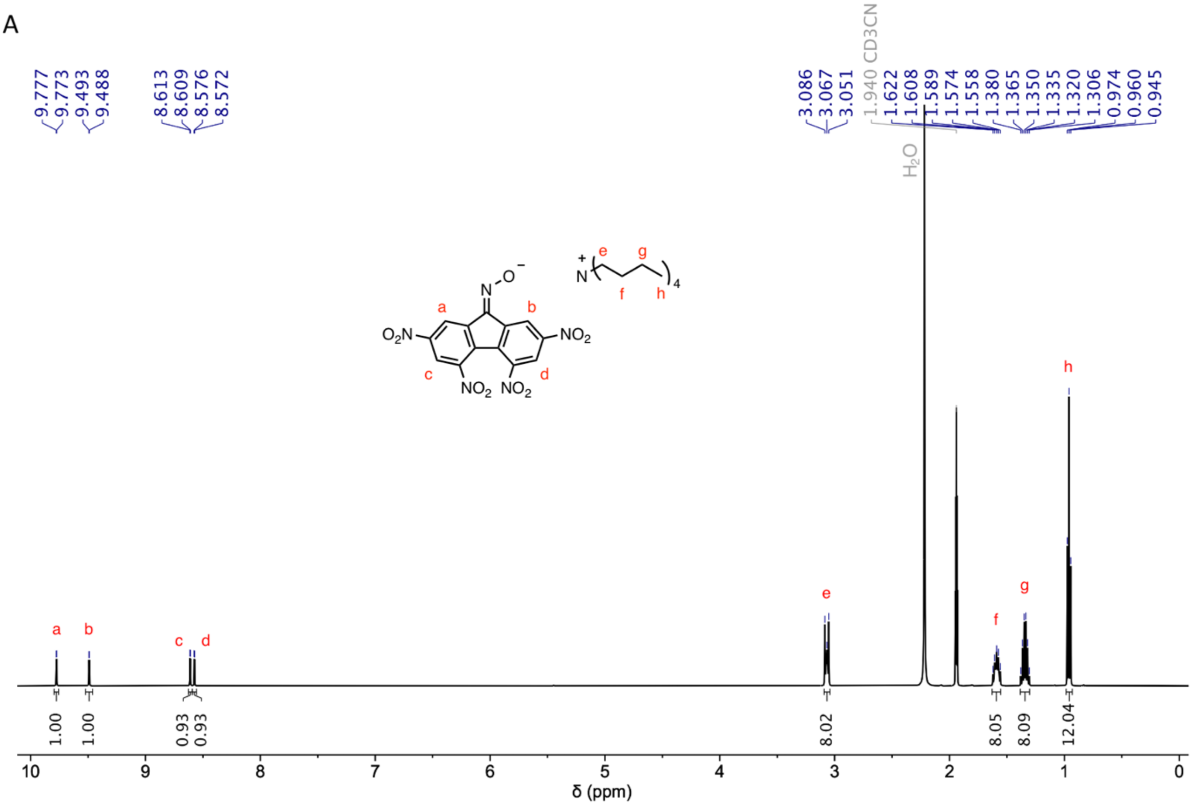

B

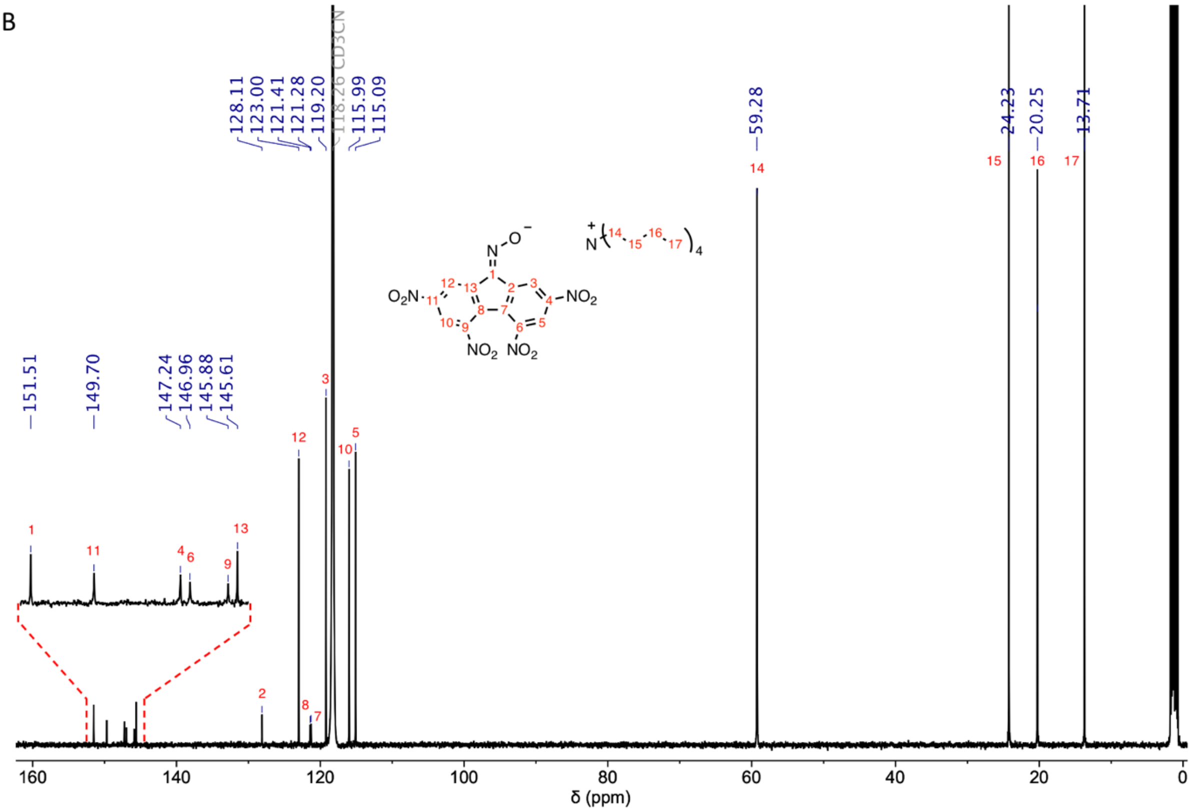

Figure S11. A) ^{1}H NMR (MeCN-d_3, 500 MHz, 298 K) and **B)** ^{13}C NMR (MeCN-d_3, 125 MHz, 298 K) spectra of compound [Bu$_4$N][**1**].

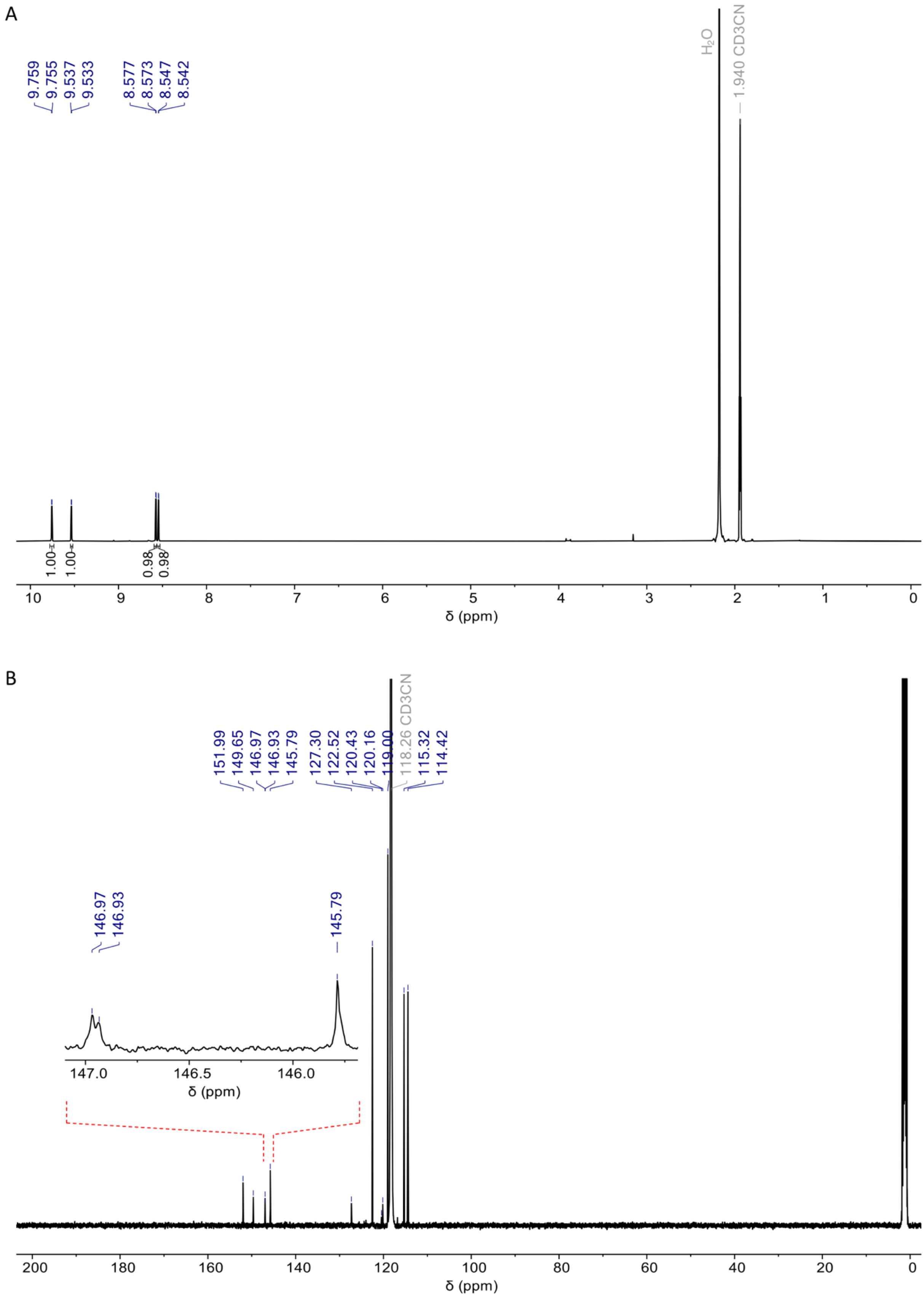

Figure S12. A) ^{1}H NMR (MeCN-d_3, 500 MHz, 298 K) and **B)** ^{13}C NMR (MeCN-d_3, 125 MHz, 298 K) spectra of compound [Na][**1**].

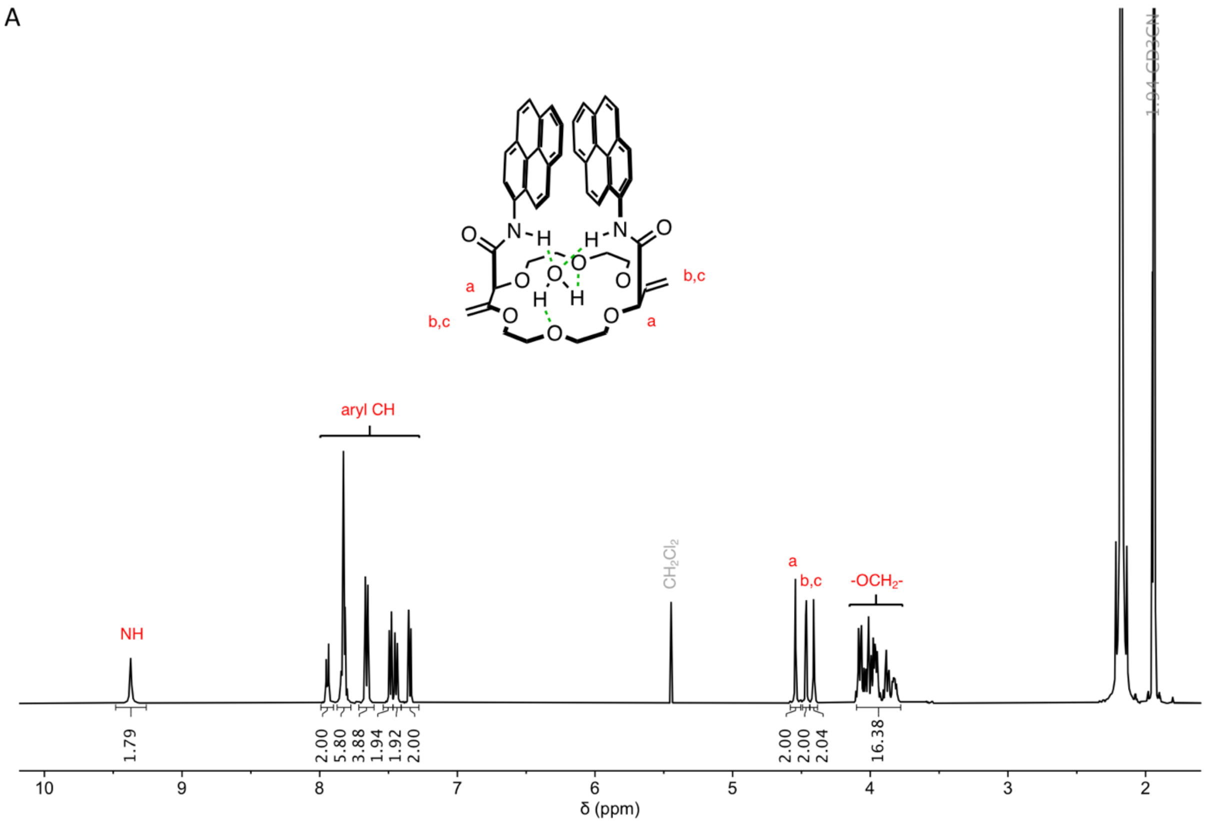

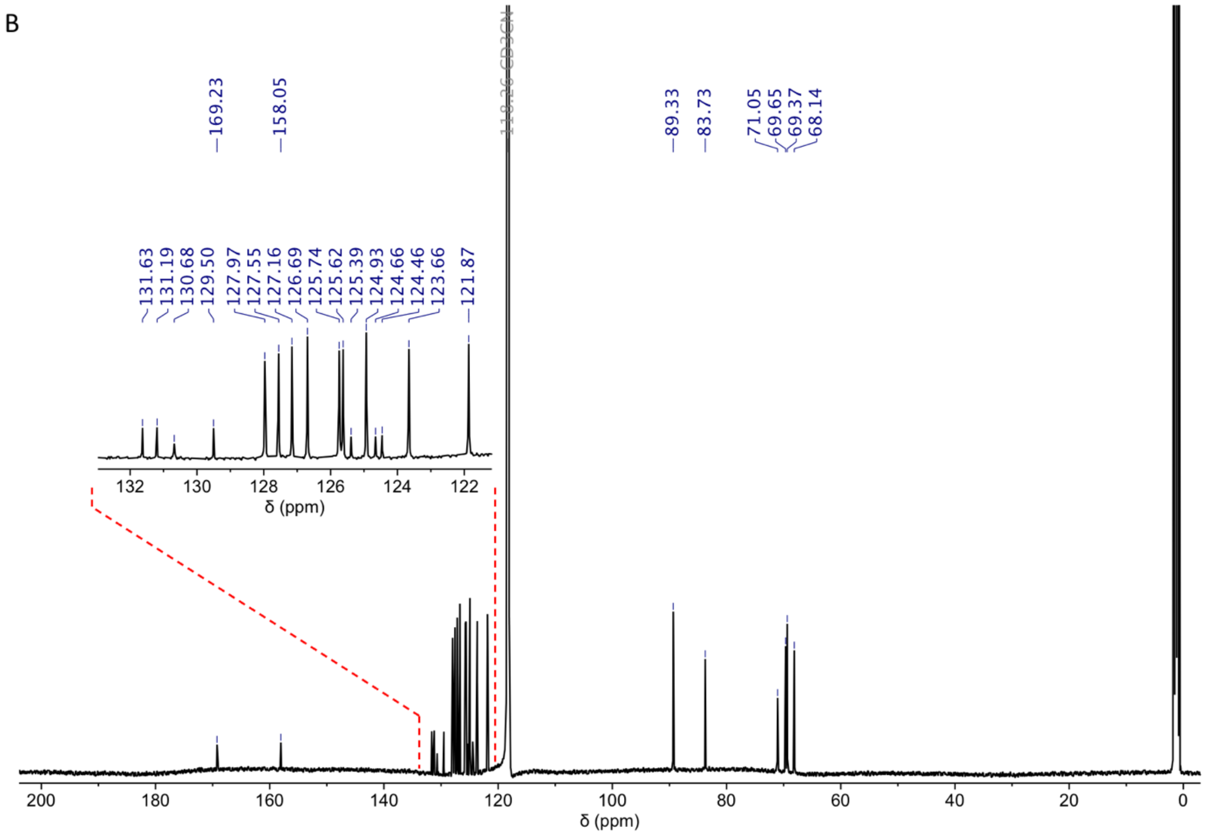

Figure S13. A) ^{1}H NMR (MeCN-d_3, 500 MHz, 298 K) and **B)** ^{13}C NMR (MeCN-d_3, 125 MHz, 298 K) spectra of compound **2**.

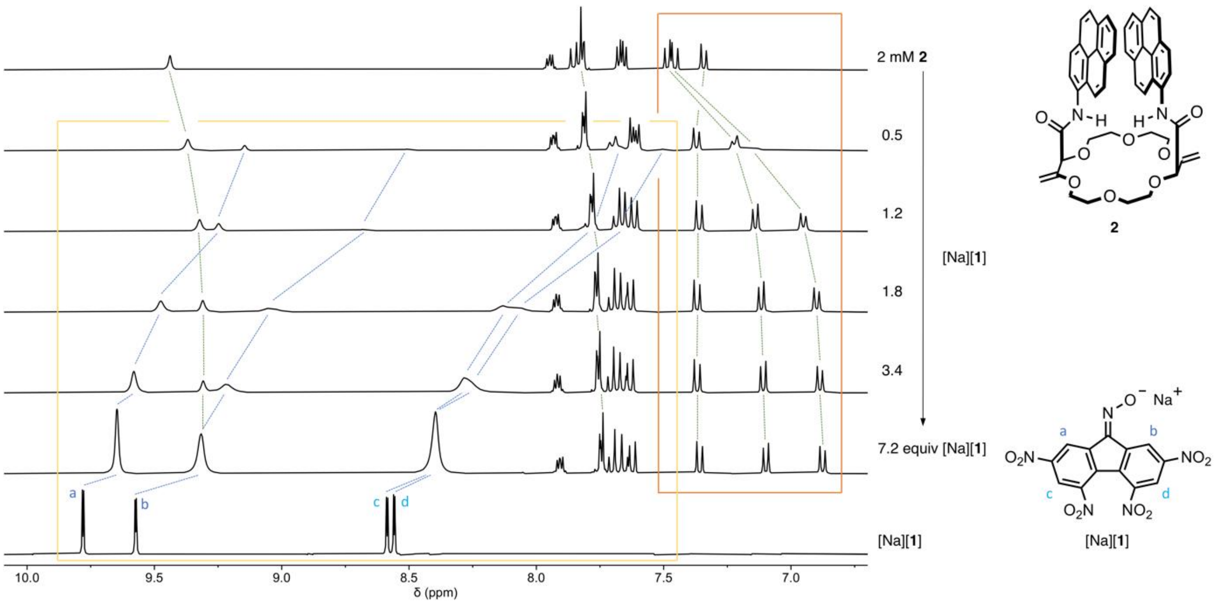

Figure S14. ^{1}H NMR (MeCN-d_3, 400 MHz, 298 K) spectra upon titration of **2** (2 mM) with [Na][**1**].

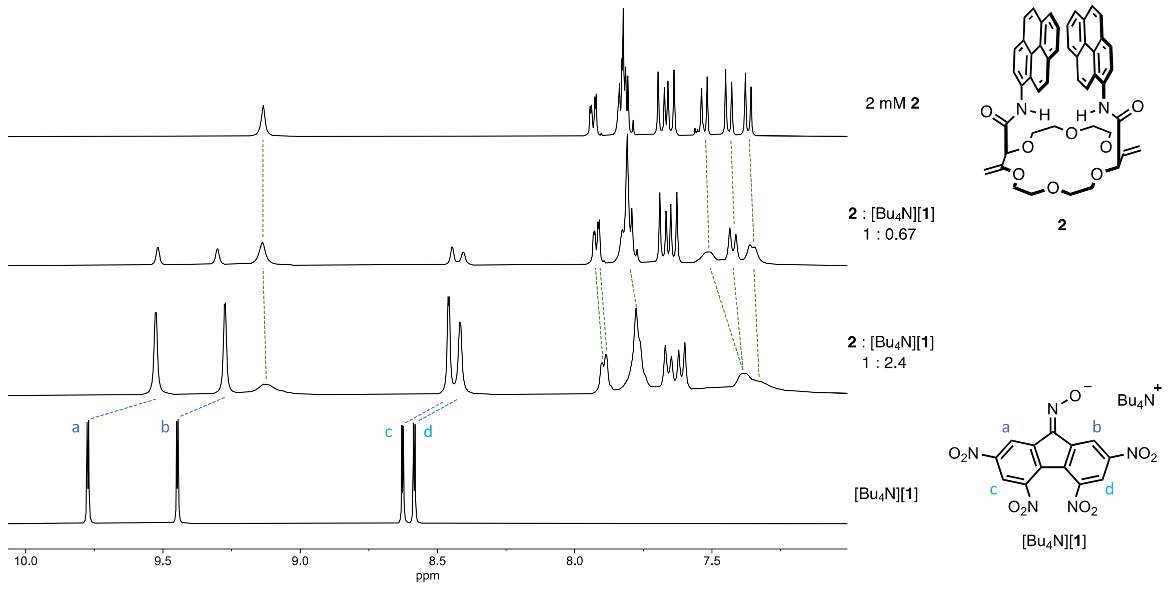

Figure S15. ^{1}H NMR (MeCN-d_3, 400 MHz, 298 K) spectra upon titration of **2** (2 mM) with [Bu_4N][**1**].

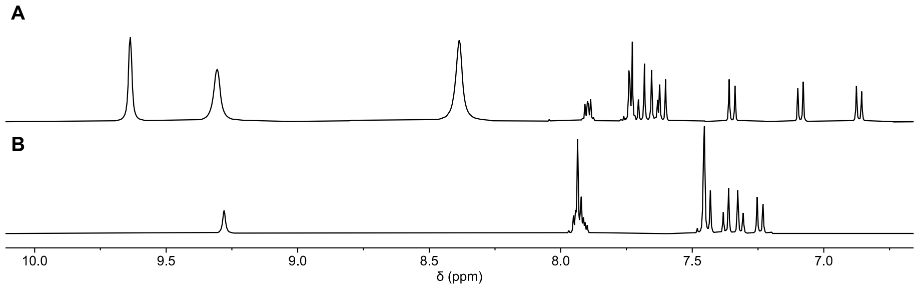

Figure S16. ^{1}H NMR (MeCN-d_3, 400 MHz, 298 K) spectra of **A)** the ternary complex *i.e.* **2** (2 mM) in the presence of 7.2 equiv [Na][**1**] and **B)** **2**•Na^+ *i.e.* **2** (2 mM) in the presence of 4.0 equiv $NaClO_4$.

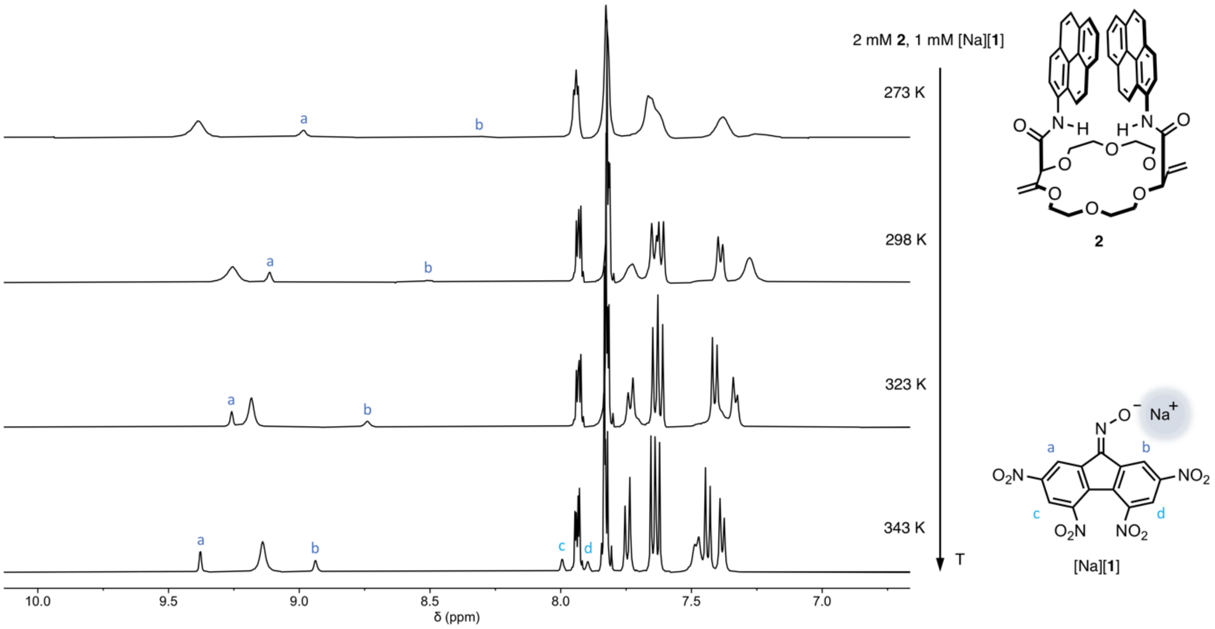

Figure S17. Variable temperature ^{1}H NMR (MeCN-d_3, 500 MHz) of 1:2 mixture of [Na][**1**] (1 mM) and **2** (2 mM).

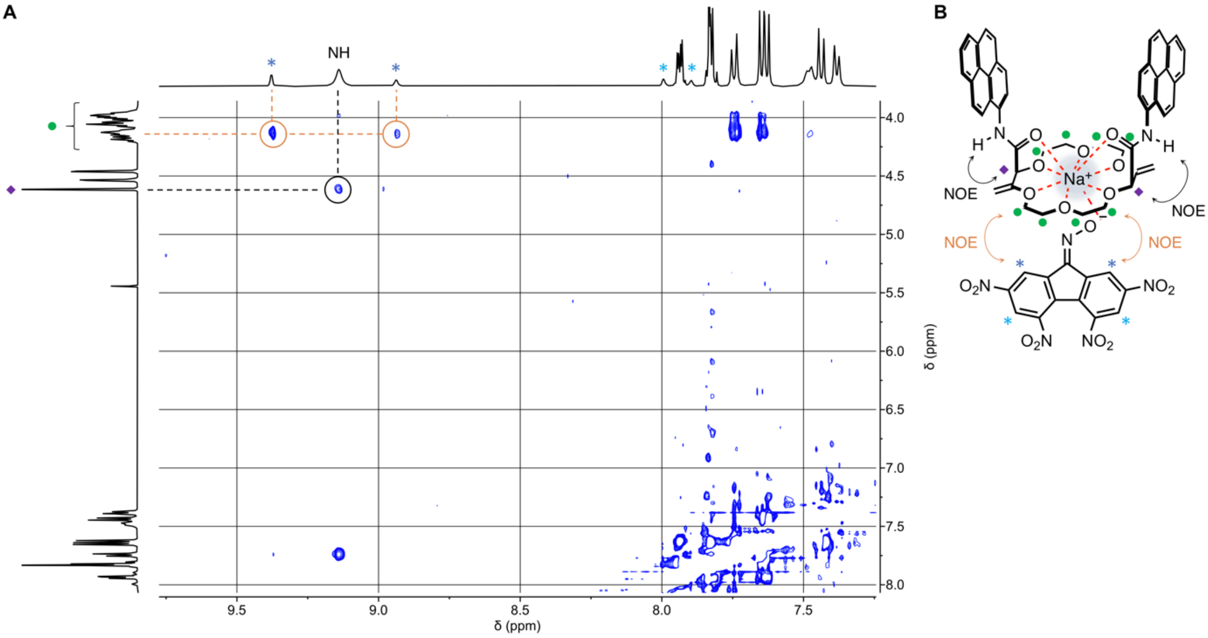

Figure S18. A) NOESY (MeCN-d_3, 500 MHz, 343 K, d_8 = 500 ms) of [Na][**1**] (1 mM) and **2** (2 mM), **B)** one of the plausible conformations in accordance.

6. Temperature-dependent equilibrium between ternary complexes

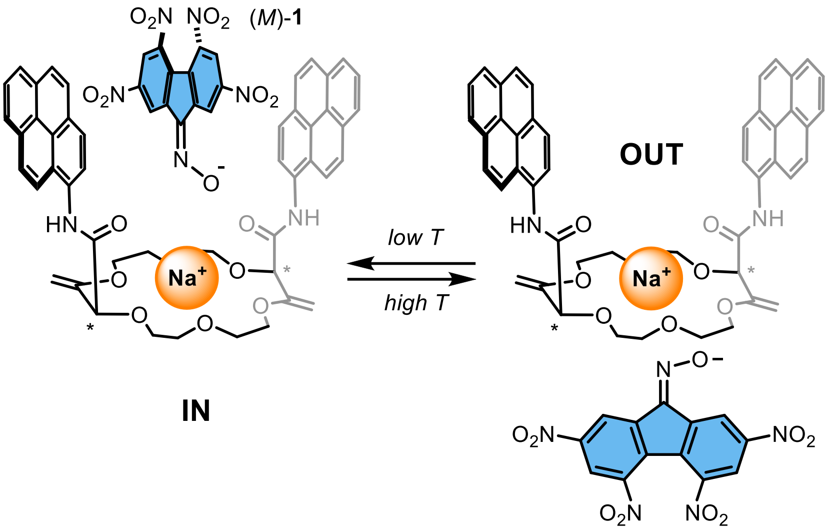

Figure S19

7. References

S1. D. Pál, C. Besnard, A. de Aguirre, A. I. Poblador-Bahamonde, G. Pescitelli, J. Lacour *Chem. Eur. J.* **2023**, e202302169.

S2. Z. Jarolímová, M. Vishe, J. Lacour, E. Bakker *Chem. Sci.* **2016**, *7*, 525–533.

S3. M. S. Newman, W. B. Lutz *J. Am. Chem. Soc.* **1956**, *78*, 2469–2473.

S4. S. B. Strashnova, O. V. Avramenko, A. V. Churakov, V. S. Sergienko, O. V. Koval'chukova, B. E. Zaitsev *Russ. J. Inorg. Chem.* **2008**, *53*, 1586–1589.

S5. D. Poggiali, A. Homberg, T. Lathion, C. Piguet, J. Lacour *ACS Catalysis* **2016**, *6*, 4877–4881.

S6. W. Zeghida, C. Besnard, J. Lacour *Angew. Chem. Int. Ed.* **2010**, *49*, 7253–7256.

S7. A. Homberg, J. Lacour *Chem. Sci.* **2020**, *11*, 6362–6369.
